# Deterministic and Empirical Approach for Millimeter-Wave Complex Outdoor Smart Parking Solution Deployments †

**DOI:** 10.3390/s21124112

**Published:** 2021-06-15

**Authors:** Fidel Alejandro Rodríguez-Corbo, Leyre Azpilicueta, Mikel Celaya-Echarri, Peio Lopez-Iturri, Ana V. Alejos, Raed M. Shubair, Francisco Falcone

**Affiliations:** 1 School of Engineering and Sciences, Tecnologico de Monterrey, Monterrey 64849, Mexico; fidel.rodriguez@tec.mx (F.A.R.-C.); mikelcelaya@tec.mx (M.C.-E.); 2 Department of Electric, Electronic and Communication Engineering, Public University of Navarre, 31006 Pamplona, Spain; peio.lopez@unavarra.es (P.L.-I.); francisco.falcone@unavarra.es (F.F.); 3 Institute of Smart Cities, Public University of Navarre, 31006 Pamplona, Spain; 4 Department of Teoría de la Señal y Comunicación, University of Vigo, 36310 Vigo, Spain; analejos@uvigo.es; 5 Department of Electrical and Computer Engineering, New York University (NYU) Abu Dhabi, Abu Dhabi 129188, United Arab Emirates; raed.shubair@nyu.edu

**Keywords:** propagation modeling, millimeter wave, 3D Ray Launching, smart parking, vegetation environment, vehicular communications

## Abstract

The characterization of different vegetation/vehicle densities and their corresponding effects on large-scale channel parameters such as path loss can provide important information during the deployment of wireless communications systems under outdoor conditions. In this work, a deterministic analysis based on ray-launching (RL) simulation and empirical measurements for vehicle-to-infrastructure (V2I) communications for outdoor parking environments and smart parking solutions is presented. The study was carried out at a frequency of 28 GHz using directional antennas, with the transmitter raised above ground level under realistic use case conditions. Different radio channel impairments were weighed in, considering the progressive effect of first, the density of an incremental obstructed barrier of trees, and the effect of different parked vehicle densities within the parking lot. On the basis of these scenarios, large-scale parameters and temporal dispersion characteristics were obtained, and the effect of vegetation/vehicle density changes was assessed. The characterization of propagation impairments that different vegetation/vehicle densities can impose onto the wireless radio channel in the millimeter frequency range was performed. Finally, the results obtained in this research can aid communication deployment in outdoor parking conditions.

## 1. Introduction

With the hope of guiding our future towards greater sustainability, the use of recent advances in communication technologies can support the dream of realizing real smart cities. The growing pattern of passenger and freight transportation has evidenced that the current transport service structure is limited in many countries [1]. In this sense, different works in the literature have presented optimization models considering all tactical-level decision problems, to maximize the obtained gains from and sustainability of transportation services [2,3,4]. At the same time, there has been an active debate about the use of delivery drones and drone passenger transportation systems as current emerging technologies for achieving improvements in mobility transportation. Reference [5] presents a systematic literature review for the socio-technical deliberations related to this topic, where it is shown that the discussion principally comprises technical and regulatory assessments. In these envisioned environments, technology and creativity come together with the aim of making society more efficient and sustainable. As part of this goal, intelligent transportation systems (ITS) will employ sensing, analysis, and control of entities in the vehicular environment (e.g., vehicles, traffic signs, parking lots or route controllers) to make transportation more efficient and secure. Considering that, by 2050, about 68% of the world’s people will live in cities [6], it is clear that ITS will be essential within the main structure of smart cities.

With the advent of autonomous vehicles within this ITS context, the tasks of driving and operating vehicles will be less and less in the hands of the human driver. At that point, fully automated vehicles will communicate wirelessly with other vehicles (V2V) or infrastructures (V2I) as a way of gathering the information necessary to act under different conditions, environments, scenarios and constraints. In this sense, the demands for higher capacity/bandwidth and low latencies in communication systems will increase dramatically each year, with predictions for future smart cities placing more stress on these aforementioned demands. In the autonomous vehicular environment domain, the need for data access in real time, raw video feed information, and real-time route control, as well as high-throughput and time constraints, has led to the proposal of millimeter-wave (mmWave) frequency bands as a way to tackle these requirements. The frequency spectrum in mmWave bands provides a large bandwidth allocation for communications systems, achieving higher throughput and lower latencies. Despite these performance advantages, communication in the mmWave frequency range presents high penetration losses and poor diffraction, which imposes many challenges with respect to its implementation. Propagation characteristics in mmWave bands are significantly different from those in micrometric frequencies, thus leading to an increase in both academic and industry interest. At the same time, mmWave frequency bands have also been considered in the vehicular environment for ITS solutions [7,8]. Characterized by low antenna heights and high mobility, the vehicular environment is highly complex in general; this fact, added to the disadvantages of using mmWave frequency bands, has recently opened a demanding research area. Therefore, it is undoubtedly essential to precisely characterize communications in these challenging high-frequency bands, especially under the effects of non-line of sight (NLOS), where the drawback of the absence of strong multipaths with respect to mmWave propagation may result in a loss of the communication link. It must be noted that mmWave radio signal shadowing can induce high losses in the received signal strength, which will be further aggravated by the use of directional beams, as is expected for mmWave Multiple Input Multiple Output (MIMO) communication systems using beamforming techniques. Regarding the study of blockages in mmWave frequency bands, particularly in the autonomous vehicular context, the most common study cases relate to blocking by other vehicles or buildings [9,10]. Additionally, obstruction by vegetation has been well documented in both the sub 6 GHz and mmWave regions, but is usually evaluated under typical environments like rural, urban, and highway areas [11,12,13,14]. In this regard, fewer works have been reported focusing on other less common scenarios and circumstances such as parking lots, tunnels, and roundabouts, where vehicle density or blockage and the presence of vegetation can influence wireless communication links.

In this work, the impact on the mmWave frequency band caused by vegetation and vehicle density is assessed in an outdoor parking lot aided by an in-house-developed 3D Ray Launching (3D-RL) algorithm. In contrast with the previous analysis presented in [10], which provided channel characterization in a typical urban environment for a combination of different cases considering obstruction between the transceivers of vehicles of distinct sizes, the aim of this work is to provide a comprehensive analysis of the propagation phenomena and radio frequency (RF) channel impairments resulting from the presence of different vehicles and vegetation densities in an outdoor smart parking scenario. With this goal, and distinguishing itself from [10], a different type of environment is considered—indeed, a complex heterogenous environment (outdoor smart parking with inhomogeneous vegetation microenvironment)—from the one presented in [10], which was a traditional urban environment (traditional urban road with traffic, buildings and people). The specific RF signal characteristics in terms of radio propagation are unique for each type of environment, and must be analyzed independently. Validation of the algorithm is achieved by performing empirical measurements in a real scenario in the 28 GHz frequency band. Discussion of the influence of vegetation and vehicle density is presented, showing that the obtained results can assist in the adequate assessment of the wireless mmWave communication channel for new vehicular communications networks, applications and deployments, when considering outdoor conditions.

## 2. Smart Parking Wireless Channel Characterization Literature Review

Although there are already so-called smart parking lots, it is only with the arrival of fully autonomous vehicles that these spaces will achieve their full potential. The requirements of autonomous vehicles in an intelligent parking environment are closely related to the demands of automated driving on a daily basis: utilities such as multiple sensors, raw camera data, and the processes of interaction between V2V and V2I communication links. Scenarios such as smart parking lots have been addressed in the literature at micrometric frequencies in works such as [15,16]; in these studies, empirical approaches were followed, showing the obtained measurement results in two indoor parking areas. In [15], an indoor parking garage scenario was evaluated considering V2V communications, providing path loss, delay spread, and small-scale fading results. Parameters for a tapped delay line model were presented and summarized. Another 5 GHz band V2V communication system in a parking garage scenario was presented in [16], where propagation path loss and root-mean-square delay spread (RMS DS) for both same-floor and floor-to-floor propagation conditions were drawn. Path loss exponents below 2 were obtained under the line of sight (LOS) case conditions, and between 2.7 and 3 for NLOS case conditions, with RMS DS results ranging from 34 to 88 ns and from 80 to 193 ns, respectively. Regarding the mmWave frequency range, the work presented in [17] analyzed the path loss (PL) in an indoor parking environment at frequencies of 26.5 GHz and 38.5 GHz for V2V communication scenarios. It is stated in this research that mmWave takes advantage of a full parking scenario and the nearby vehicles, resulting in a cumulative decrease in PL. In the literature, outdoor parking environments have less frequently been considered; these are usually characterized by large open areas, often with abundant and inhomogeneous vegetation, which is challenging in terms of channel modeling and propagation characterization. In [18], the path losses in an outdoor parking scenario at 28 GHz and 38 GHz were inspected, employing a measurement campaign and the adjustment of PL to the close-in model (CI) free space reference, and providing a floating-intercept (FI) PL model.

The aforementioned propagation impairments due to the presence of vegetation in outdoor parking environments have a great impact on communication channels, giving rise to potential increases in the probabilities of NLOS, and temporal and angular dispersion parameters [12]. The use of barriers with abundant vegetation in outdoor parking environments is a common practice for the purposes of safety, security and scenery, usually separating different parking instances. Under these conditions, V2I communication may be affected by high attenuations and large detriments in the temporal parameters of the communication link. The effect of foliage has been well documented in recent years, and several models have been proposed, such as the ITU 833-9 model [19], Weissberger [20], COST235 [21], and Fitted ITU-R (FITU-R) [22]. These models fall under the category of modified exponential decay (MED) vegetation loss models, and they are relatively simple and effortless to implement. In [13], a 28 GHz foliage attenuation effect in seasonal changes was assessed under a directional antenna pattern. This work presents a model based on the MED approach, which also provides spatial–temporal channel characteristics like delay spread, coherence bandwidth, and angular spread analysis. Other research works following these foliage recommendations can be found in [14,23,24,25,26]. Specifically with respect to the use of deterministic modeling techniques like Ray Tracing, some works that use this approach to evaluate foliage effects include [27,28]. Another large-scale PL model that has been widely used is the CI free-space reference distance model. In reference [12], empirical results were presented across the 28 GHz and 39 GHz bands, and the CI model was applied to characterize directional and omnidirectional PL in vegetated areas.

MED models usually depend on information from the environment like vegetation depth, although this parameter may not have a significant effect in a single-line tree barrier perpendicular to the transmitter. Under this condition (as may be the case in an outdoor parking lot), the recommendation may be to use a blockage model for a single tree obstruction, but the variability in vegetation density may affect scattered signals. Likewise, vehicle density may change during day/night hours, and this may impact the received signal for in-site vehicles. As an example, in reference [16], the authors detected changes in parameters like the PL exponent and the RMS DS when considering different vehicles densities.

From the perspective of the channel modeling approach, accounting for vegetation and vehicle density can take several forms. Typically, non-geometric stochastic models (NGSM) use numerous measurement campaigns in diverse environments, extracting channel parameters that reflect the conditions under consideration. These conditions may be vegetation at the side of the road [29] or different traffic densities [30,31]. Conversely, in geometric models, accounting for vegetation and vehicle density and obstruction can be performed natively, as long as it is considered in the model layout. With respect to the geometric stochastic models (GSM) approach, reference [32] employs the geographical features of the simulated environment to divide network communication links into three different groups: LOS, NLOS due to vehicles (NLOSv), and NLOS due to buildings/foliage (NLOSb). Using a deterministic approach, the Ray Tracing modeling technique also computes these effects natively, and relatively more complex representations of both vehicles and vegetation can be used, at the expense of higher computational cost [33].

In general, the impact of vegetation and vehicle density on the mmWave frequency range is still an issue that needs further investigation. The effects of vegetation density in wireless channel characteristics are pivotal to precisely assessing the propagation channel impairments in complex outdoor environments such as parking lots. Likewise, changes in vehicle density in outdoor parking scenarios may introduce aggravated large-scale and temporal dispersion effects in the communication channel. Considering all these statements, further studies on the mmWave frequency bands are required.

## 3. Materials and Methods

As commonly found in outdoor parking lots, trees planted in divided parking areas serve both as decoration and to provide shade. These trees’ barriers are frequently composed of a single line of trees and shrubs, usually equally spaced. Additionally, the density of parked vehicles may change during the day/night hours, consequently changing the wireless propagation channel characteristics in such types of environments. To assess the channel impairments and propagation effects, an outdoor parking lot placed within the Monterrey campus at Tecnologico de Monterrey was considered. The scenario was modeled in 3D and simulated in an in-house-developed deterministic RL algorithm. In addition, measurements were performed in the real scenario, providing empirical validation of the results from the simulated datasets. The sections introduced below describe the simulation approach followed in the 3D-RL algorithm, as well as the measurement campaign carried out in the real scenario.

### 3.1. Simulation Description

**Figure 1:**
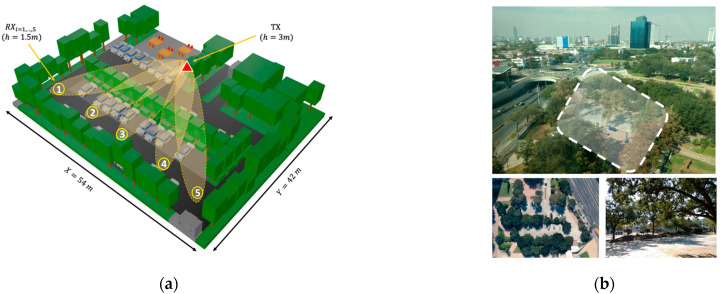
General and detailed view of the simulated 3D scenario: (**a**) rendered view of the scenario; (**b**) real scenario photos with the consideration of scatterers as vegetation and vehicles, among others.

**Figure 2:**
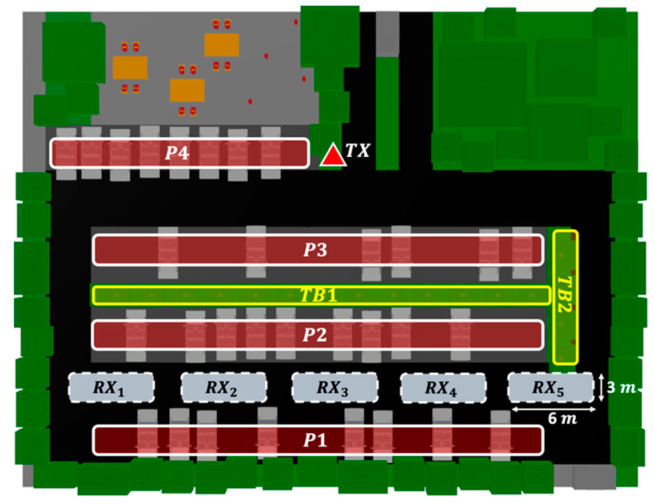
Schematic view of the reception points (RX1 to RX5) and the affected areas: vegetation (TB1 and TB2) and parked vehicles (P1 to P3).

Different approaches can be considered for analyzing the wireless communication channel within an outdoor smart parking. On the one hand, empirical approaches are rapid and easy to use, but their main drawback lies in their validity for environments other than that in which the measurements were performed [34]. On the other hand, full wave techniques are the most accurate approaches, with the principal disadvantage of inherent computational time for large and complex environments, such as an outdoor parking lot, being unaffordable for these types of environments [35]. As a mid-point, methods based on geometry-based deterministic approaches achieve a trade-off between the accuracy of the results and the computational time required for the simulation [36]. Based on this, an in-house deterministic 3D-RL algorithm was employed to assess the vegetation and vehicle density impact in the 28 GHz frequency band for the deployment of smart parking solutions. Detailed information regarding the 3D-RL algorithm and its validation in vehicular environments can be found in [37,38,39,40]. The modeled scenario is a real outdoor parking lot at Tecnologico de Monterrey Campus in Monterrey, Mexico. It was selected due to its similarity to typical outdoor parking scenarios with abundant inhomogeneous vegetation. The stage dimensions are 54 m length by 42 m width, and a maximum height of 15 m was considered in the virtual representation. In Figure 1, the considered scenario for simulation is presented, as well as the real parking lot, characterized by abundant vegetation in the surroundings, and a vegetation dividing barrier typical of outdoor parking lots. The trees and shrubs of the barrier are distributed with uniform spacing, taking into account the different densities of vegetation that will be simulated. The considered barrier is composed of trees with a height of 6.2 m and shrubs with a height of 1.2 m, emulating the real study case. The designated areas for parking vehicles are presented in Figure 2, identified as P1 to P4, where typical personal vehicle cars were modeled in these areas for the purposes of the simulation.

For the purposes of the simulation, a transmitter antenna placed in a streetlight at a height of 3 m was considered, as well as several NLOS challenging receiver locations in the scenario. Different directional beams were assessed (represented in Figure 1a for reference), aiming at points located on a typical vehicular receiver (at 1.5 m height) on the other side of the vegetation barrier according to the arrangement presented in Figure 2. Five reception points were simulated under different vehicle and vegetation density conditions. It must be remarked that only the density of one of the study cases was modified at a time, while the other was kept at a constant average density of 50%, in order to clearly identify its affect in the wireless communication channel.

The area occupied by the tree barrier, as shown in Figure 2 (TB1 and TB2), is approximately 104 m². Taking into account the characteristics of the trees and shrubs, and using the entire area provided for vegetation, a total of 17 trees and 17 shrubs were employed for the maximum density (100%). From this maximum density and taking into account the uniformity of the vegetation distribution, three other scenarios were simulated for 75, 50, and 25% vegetation densities. Regarding the considered designated parking areas, car density distributions were altered only in the P1 to P3 parking areas (shown in Figure 2), due to a direct impact on the wireless communication link. Conversely, P4 was not altered, as it does not provide a direct obstruction in the wireless link. Taking into account that each area can house a maximum of 15 cars, the maximum density of the vehicles in these considered areas (P1–P3) was 45 cars (100% vehicle density study case). In this sense, the simulations were performed for 100, 75, 50, and 25% of the total vehicular car density. The vehicle locations in the different density study cases were permuted randomly and uniformly distributed in all possible combinations. Table 1 presents the most relevant parameters used in the simulations.

### 3.2. Measurement Campaign Description

**Figure 3:**
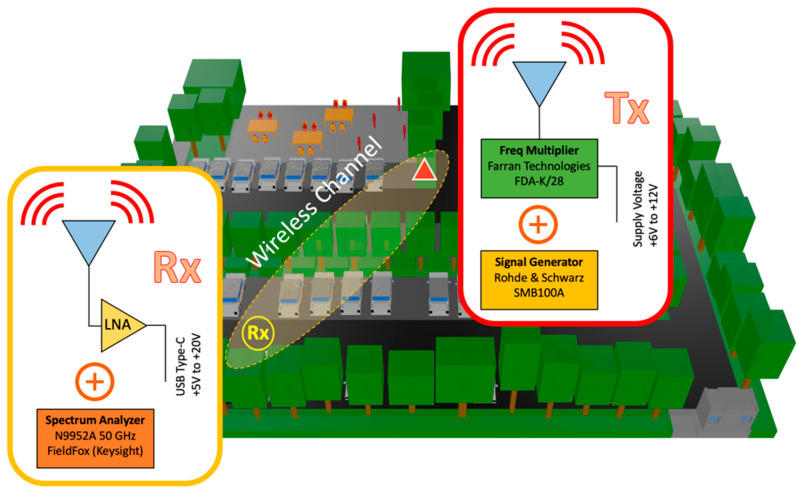
Schematic scenery of the experimental setup in the considered outdoor parking lot.

**Figure 4:**
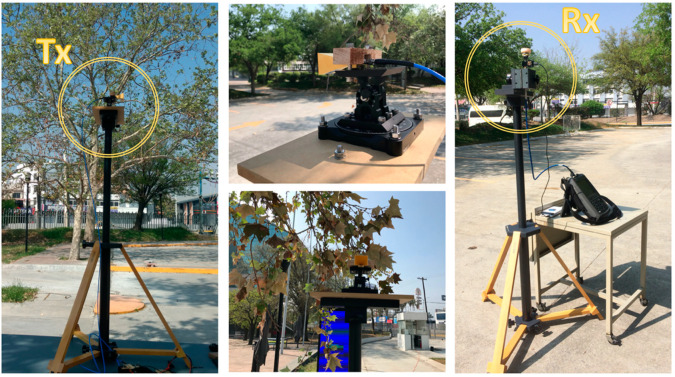
Transmitter and receiver experimental setup in the actual outdoor parking during the measurement campaign.

To validate the proposed simulation methodology, an experimental measurement campaign was designed and developed in the actual scenario situated in one of the parking areas of Tecnologico de Monterrey, Campus Monterrey. The selected area is shown in Figure 1b, with an aerial view and a detailed image of the tree vegetation barrier located within the parking lot. A transmitter was placed in the same position as shown in Figure 2, at a height of 2.35 m, and measurements were obtained at the same receiver points $RX_{i=1,\ldots ,5}\;$ as those presented in Figure 2, at a height of 1.5 m. For the transmitter, an SMB100A Rohde & Schwarz signal generator and an FDA-K/28 frequency multiplier from Farran Technologies were used to transmit the signal at the 28 GHz operating frequency. The transmitter antenna was a Ka-band pyramidal horn antenna Model SAR-2013-28KF-E2, from SAGE Millimeter, Inc. (Torrance, CA, USA), with 20 dBi nominal gain and typical half power beamwidth of 14 and 16 degrees on the E-plane and H-plane, respectively. It was directed towards the receivers’ points with the aid of an antenna positioner from Thors Labs. For the receiver, a N9952A 50 GHZ FieldFox portable spectrum analyzer from Keysight Technologies was used with a Ka-band omnidirectional antenna Model SAO-2734033045-KF-C1-BL from SAGE Millimeter, Inc., connected to a low-noise amplifier (LNA) at 30 dBi. A dielectric tripod (AT-812 Antenna Tripod from Com-Power Corporation (Brea, CA, USA)) was used to place the transmitter and receiver antenna at the measurement points. Figure 3 shows the block diagram of the equipment deployed for the transmitter and receiver parts in the performed experimental measurement campaign. A collage of different images for the transmitter and receiver part during the measurement campaign in the real scenario is presented in Figure 4, where the vegetation surroundings can be appreciated. It must be pointed out that the measurement campaign was performed during the SARS-CoV-2 pandemic, and due to this fact, the parking area of the university campus was closed to vehicles, so measurements were performed in a controlled area where neither people nor vehicles were moving in the surroundings. In this sense, the tree barrier vegetation impact was able to be more accurately characterized.

## 4. Simulation Results and Discussion

### 4.1. Path Loss

**Figure 5:**
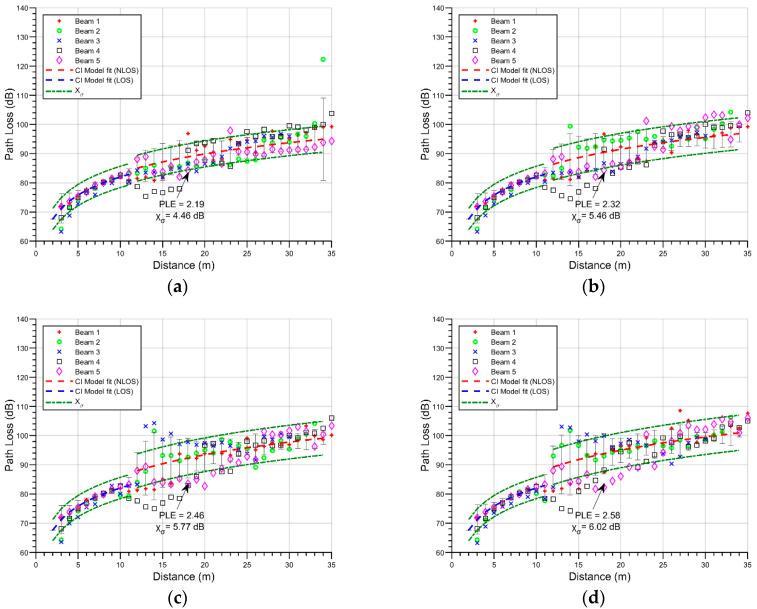
Path loss values for all beams and CI model fit: (**a**) 25% vegetation density scenario; (**b**) 50% vegetation density scenario; (**c**) 75% vegetation density scenario; (**d**) 100% vegetation density scenario.

As stated previously, the impact of variable vegetation and vehicle densities in an outdoor parking lot was evaluated with the assistance of a 3D-RL simulation approach. To assess these effects in a mmWave steerable wireless communication link, five different radiation antenna beams were considered, directed towards the free line parking, as is shown in Figure 1a. Each beam passes through a single-line tree obstruction located in the middle of the parking lot. Although the effect of a single-tree obstruction has been well studied in the literature [19,33], the net density effect of a one-tree line has not yet been considered. The path loss (PL) for every beam is obtained throughout its trajectory up to the receiver point, depicted in Figure 5, which shows the plot of every beam versus distance. As the distance between the transmitter and the tree line is approximately 11 m, every spatial point before this line was considered to be LOS, and beyond the 12-m mark, they were considered as NLOS. Figure 5 shows the PL for four different vegetation densities (25%, 50%, 75%, and 100%), while no changes in vehicle density (50%) were implemented.

To obtain an approximation to the net effect, a fitted CI model is used, which has the advantage of a frequency-dependent parametrization. The estimation of path losses using the CI model follows the following equation [41]:

(1) $$PL_{CI}\left(f,d\right)\left[dB\right]=FSPL\left(f,d\left(1m\right)\right)\left[dB\right]+10n\log_{10}\left(d\right)+X_{\sigma }$$

where $FSPL\left(f,d\left(1m\right)\right)=20\log_{10}\frac{4\pi f}{c}$ shows the free-space path loss in dB for a separation distance of 1 m between the transmitter and the receiver at a carrier frequency *f*; *c* corresponds to the speed of light, *n* is the path loss exponent (PLE), *d* determines the 3D separation distance among the transmitter and the receiver, and $X_{\sigma }$ presents the shadow fading standard deviation for large-scale signal fluctuations. As shown in Figure 5, beyond the LOS mark there is a more aggravated loss, and the shadow fading is also characterized by an increasing factor. In the case of Figure 5a, the vegetation density is set at 25%, and as a result, the mean PLE is 2.19; this trend continues in the subsequent figures, reporting values of 2.32 for a vegetation density of 50%, 2.46 for 75%, and 2.58 when 100% of the designated area is occupied by trees and bushes. The vegetation density is positively correlated with the PLE, which is related to the increase in the numbers of rays that are scattered by the trees. In the case of the shadow fading, values of 4.46 dB, 5.46 dB, 5.77 dB, 6.02 dB were obtained for vegetation densities of 25, 50, 75, and 100 percent, respectively. There is also a positive correlation between shadow fading and the distribution densities, although considering the dispersiveness of the data, this relationship may not hold true; thus, the analysis of these results is expanded in Section 3.2.

**Figure 6:**
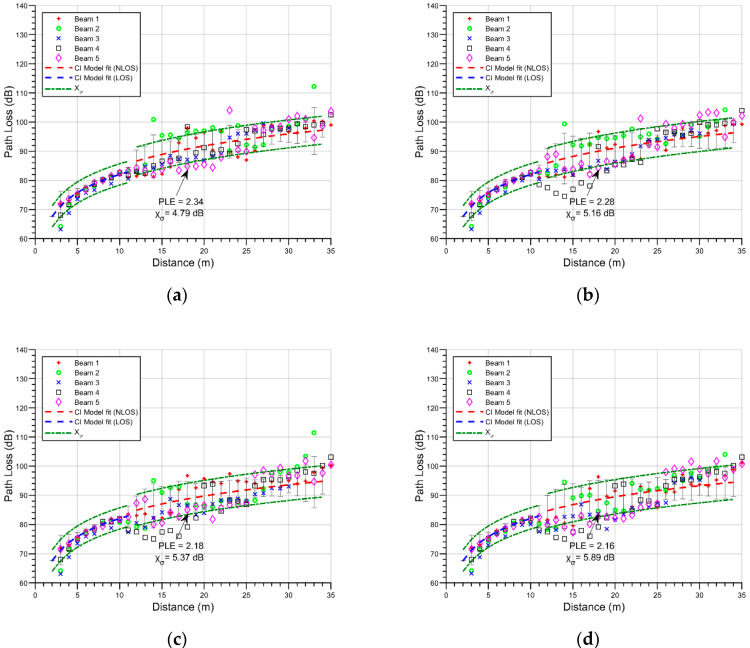
Path loss values for all beams and CI model fit: (**a**) 25% vehicle density scenario; (**b**) 50% vehicle density scenario; (**c**) 75% vehicle density scenario; (**d**) 100% vehicle density scenario.

To gain insights into the impact of changes in vehicle density in the considered scenario, Figure 6 shows the PL for all beams for density changes in the parked vehicles, while a fixed vegetation density (50%) was considered. In this stage, the PLE changes are 2.34, 2.28, 2.18, and 2.16 with vehicle density percentages of 25, 50, 75, and 100, respectively. There is a relatively low negative correlation in these results between vehicular density and the PLE. This effect has also been pointed out in other works in the literature. In [17], the PL characteristics of mmWave in an indoor parking garage environment were presented with empty and full vehicles cases. The results showed a decrease in the PL effect when a full parking scenario was used. At the same time, the work presented in [39] analyzed V2V and I2V communication links in an urban environment with different vehicular traffic densities, showing higher received signal power values for high vehicular density than for low vehicular density in the area close to the transmitter (up to approximately 30 m), while at greater distances there were no observable differences.

With respect to the shadow fading in the considered outdoor parking scenario, it also increases with vehicular density, from 4.79 dB to 5.89 dB. The absence of a strong change in the PLE is somehow to be expected, as the height of all of the simulated vehicles was 1.5 m, which is the same height as the receiver antenna. Because of this, the changes in path loss are related to destructive/constructive interference made by weaker components scattered in the nearby vehicles. The increase in shadow fading is also related to this effect, with the dispersiveness of the multipath components increasing as a result of the increasing number of vehicles.

### 4.2. RMS Delay Spread

**Figure 7:**
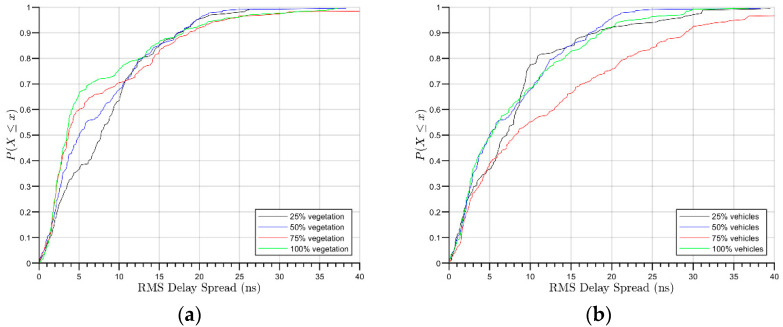
RMS DS Cumulative Distribution Function for all designated reception areas RX1 to RX5 (see Figure 2 for reference): (**a**) different vegetation densities; (**b**) different vehicle densities.

The root-mean-square delay spread (RMS DS) is defined as the second central moment of the multipath signal power delay profile (PDP), and it is a good measure of the multipath dispersiveness. This parameter is defined as [42]:

(2) $$RMS\;DS=\sqrt{\left(\bar{\tau^{2}}-{\left(\bar{\tau }\right)}^{2}\right)}$$

(3) $$\bar{\tau^{2}}=\frac{\sum_{k}P\left(\tau_{K}\right)*{\left(\tau_{K}\right)}^{2}}{\sum_{k}P\left(\tau_{K}\right)}$$

(4) $$\bar{\tau }=\frac{\sum_{k}P\left(\tau_{K}\right)*\tau_{K}}{\sum_{k}P\left(\tau_{K}\right)}$$

where $P\left(\tau_{K}\right)$ is the amplitude of the multipath component in $\tau_{K}$; $\tau_{K}$ is the relative delay of multipath k with respect to the first detectable component arriving to the receiver. In the scenario presented in this work, considering all density combinations, the RMS DS was acquired in the free lane behind the tree line, as represented in Figure 2. In all cases, the PDP for a 6- by 3-m-wide section of the road was taken for each beam. The 6-m section corresponds to the approximate width of the antenna boresight axis and the half power beam angle, and the 3-m selection is the free lane width. Although the RL algorithm has virtually an infinite Dynamic Range (DR) limit, a 25 dB DR is used in the simulations in order to remove weak multipath components into the calculations [16]. Figure 7 shows de RMS DS Cumulative Distribution Function (CDF) of all of the changes in vegetation and vehicle densities.

Figure 7a presents the RMS DS CDF in the different vegetation scenarios. The obtained mean RMS DS varies between 6 to 9 ns, and 90% of the multipath component arrives within approximately 17 to 19 ns.

Figure 5b shows the CDF for the cases in which the vegetation is maintained at 50% density and the vehicle density is changed. As seen in the figure, for this relationship, the mean RMS DS is between 7 and 12 ns. Under these scenarios, 90% of the multipath contributions arrive within approximately 17 to 18 ns in 25, 50 and 100 percent of parking spaces used, and in less than 29 ns for the 75% scenario.

### 4.3. Density Effects

**Figure 8:**
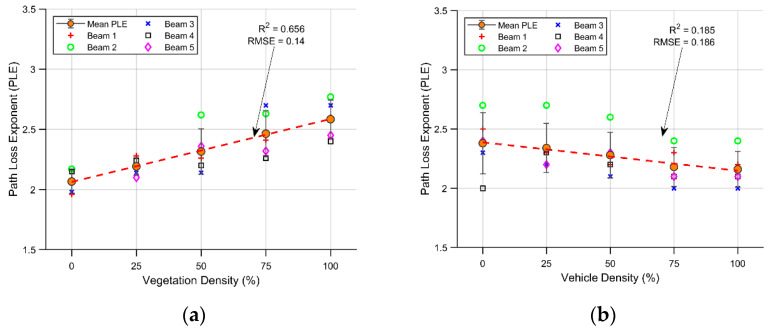
Density effects on the PLE: (**a**) different vegetation densities; (**b**) different vehicle densities.

**Figure 9:**
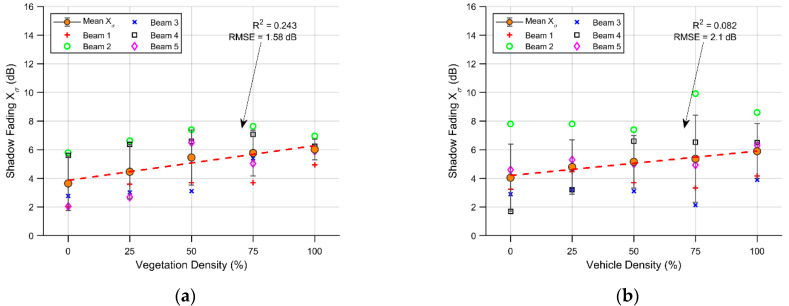
Effects of density on shadow fading: (**a**) different vegetation densities; (**b**) different vehicle densities.

As a benchmark, two more simulations were carried out, corresponding to vegetation effects of 0% and a vehicle density of 0%. Both simulations, together with the 25 to 100 percent scenarios, are presented in Figure 8 and Figure 9, including PLE and shadow fading against vegetation/vehicle density effects. Figure 8 presents the density effect over the PLE, Figure 8a specifically represents the effect of net vegetation density on this parameter. In this case, the mean PLE for all paths has a positive slope with increasing vegetation density. A linear fit is represented by the red dashed line, showing an R^2^ of 65.6% and a root-mean-square-error (RMSE) of 0.14. The variations in vegetation density represent 62.6% of the positive trend exhibited by the PLE, demonstrating a high correlation between them. These results are in alignment with the increase in the number of obstacles between the transmitter and the receiver; when a more compact barrier comes between them, a more aggravated PLE is obtained. A linear fit is represented in the figure as a way of capturing the main effect. The dispersion increase is also noticeable between low and high density, from 0 to 100 percent densities, respectively. This is the result of a nonhomogeneous increase in the scenario, from strong steady conditions for every path at low density, to high differences in trajectory conditions between one path and another at high vegetation concentrations (including gaps between the trees).

Figure 8b presents the PLE in the scenarios where the vehicle density changes, as well as the 0% vehicle scenario. In this case, the slope exhibits a negative trend, and the relation between the magnitude of the dispersion and the slope results in an R^2^ value of 18.6%, exhibiting a relatively low causal relationship between these effects. The root-mean-square-error (RMSE) is 0.186, a magnitude similar to that in the vegetation scenario. The mean PLE varies between 2.34 to 2.16 for variations in vehicle density between 0 and 100% for all usable parking spaces. As a result, the PLE is not a noticeably affected by changes in vehicle density as it is by its vegetation counterpart, although there is a negative trend. This is related to the increase in nearby reflection from parked vehicles, which contributes to a decrease in the path loss effect. Because in all cases, the vegetation line is present in the barrier at a 50% distribution, accordingly, the dispersion around the mean exhibits no noticeable changes as the vehicle density changes; as can be seen in the figure, the PLE remains close to the same trend value in almost every beam. This behavior can also be observed in the 3rd beam, where the PLE closely follows the downward trend in every scenario. This result supports the idea that in an I2V scenario, assuming regular size vehicles, the vehicle density has a relatively low impact on the PL, while this may be different under a V2V scenario.

The shadow fading obtained in the cases in which the vegetation density was altered is presented in Figure 9a. The lineal fit has a positive slope with an R^2^ of 24.3% and an RMSE of 1.58 dB. In all scenarios, the shadow fading varies from 2 dB to 8 dB, and the mean shadow fading goes from approximately 3.7 dB to 6 dB. It is noticeable that the dispersiveness around the mean shadow fading tends to decrease with the percentage of vegetation present in the scenario, this is related to an increase in the homogeneity of the barrier. In Figure 9b, the shadow fading for the different vehicle density scenarios is presented. Additionally, with a positive trend in its linear fit, with an R^2^ of 8.2% and a RMSE of 2.1 dB, the shadow fading in each path varies between 1.9 dB to 10 dB.

In both cases, the relationship between density and shadow fading is relatively weak and does not undergo any major changes. This is closely related to the events encountered by every beam, and is more affected by vegetation blocking than vehicle density.

In the case of the RMS DS in the vegetation scenarios, the relationship between the variables presents a negative correlation. This result is related to the presence of strong direct and first-order multipath components, compared to the weak components reflected by the nearby trees, which are more affected by increases in vegetation. The main RMS DS values are 8.93, 8.34, 7.45, 7.62 and 6.69 ns with an increase in vegetation density from 0 to 100 percent.

For the vehicle density scenarios, compared with their counterpart, there is a slight increase in the mean RMS DS. The RMS DS values for the 0 to 100 percent parked vehicles scenario correspond to 12.18, 8.11, 7.45, 12.24 and 7.84 ns, respectively. This difference in outcome between the vehicle and vegetation scenarios is related to the increase in metallic objects (vehicles) around the receiver as the density increases, which results in an increase in multipath contributions that influence the RMS DS.

## 5. Measurement Results

**Figure 10:**
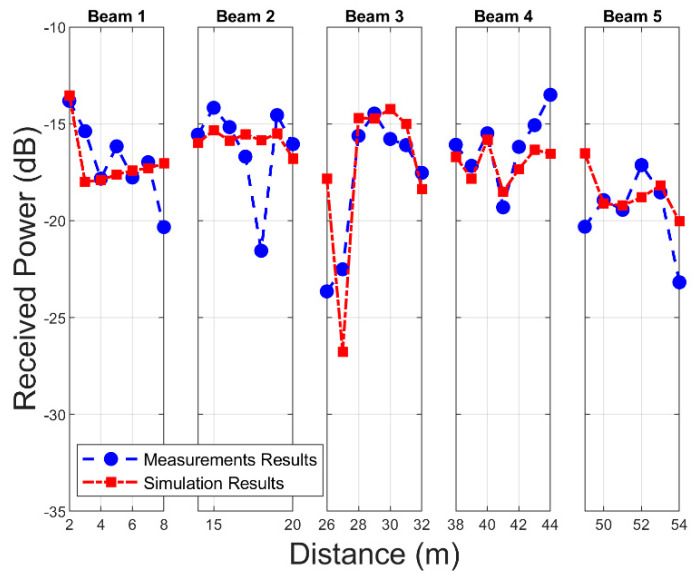
Measurement results for Beam 1 to Beam 5 in the designated locations (see Figure 2 for reference), and 3D-RL simulation results for these spatial positions.

In order to validate the in-house 3D-RL algorithm, a campaign of measurements was conducted in the outdoor parking scenario at Tecnologico de Monterrey. With this objective in mind, a virtual scenario similar to the real scenario was modeled, taking into account aspects such as the distribution and location of vegetation and vehicles. Again, aspects such as the characteristics of the environment, small and large objects, and the electromagnetic characteristics of the materials were meticulously emulated in order to capture the particularities of the scenery. The reception points were located on the free road that passes on the other side of the vegetation barrier, in this respect, the measurement campaign and the simulation determined the received power in linear sections with a length of 6 m along the road (as represented in Figure 2). Figure 10 presents a comparison of the received power between the measurement campaign data and the 3D-RL simulation results for the different beam sections.

The average received power throughout this stage in the measurement campaign was −17.20 dB, ranging between approximately −16 and −19 dB in each evaluated section. In the case of the simulation, this parameter was −17.14 dB, maintaining agreement with the real scenario. In this regard, the RMSE for each measured section with respect to the simulation was 1.69, 2.3, 2.86, 1.4, and 1.6 dB for beams 1 to 5, respectively. Although the main characteristics of the scenario were considered in the simulation, the simplification of the objects and their approximate positions within the scenario are parameters that could have influenced the results. Despite this, the total RMSE was approximately 2.07 dB, an acceptable margin that validates the 3D-RL algorithm.

## 6. Conclusions

In outdoor parking scenarios, the use of barriers with abundant vegetation is common practice. In addition to being a less widely studied environment, outdoor parking lots may contain many different vegetation configurations, as well as different densities of parked vehicles during daylight hours. Considering the influence of RF and the effects of these particularly challenging scatterers with respect to the implementation of smart parking solutions in this type of complex outdoor environment, an adequate wireless channel characterization is pivotal for providing full network coverage, vehicular communication services, and system capabilities at mmWave frequency bands. In this work, we present a PL and temporal dispersion analysis considering different changes in the densities of vegetation and vehicles in an outdoor parking scenario in the 28 GHz frequency band, allowing a deep understanding of the wireless communication channel and the possible limitations and constraints of a proper smart parking solution development/deployment under real conditions. On the basis of the obtained results, some relevant conclusions can be drawn:

The fitted parameters to the CI model for each analyzed case yield a causal change in the PLE with respect to variations in vegetation density. In this regard, the PLE obtained for the minimum density was 2.07, reaching a maximum of 2.58 at 100% vegetation density.

With respect to vehicular density, the effect on the PLE was less evident, ranging between 2.34 and 2.16.

With regard to the shadowing effect, the same increasing trend as that observed with respect to vegetation and vehicular density was evidenced.

Another aspect analyzed was temporal dispersion, for which the RMS DS values were extracted on the path of a receiver obstructed by vegetation. Regarding the changes in vegetation density, the mean RMS DS was between approximately 6 and 9 ns for all cases, and for the parked vehicles scenarios, this parameter was between approximately 7 and 12 ns. In general, the RMS DS was influenced far less by the different scenarios, displaying a negative trend with increasing vegetation.

As validation and support for the 3D-RL algorithm, a complete measurement campaign was carried out in a real outdoor scenario, located on the Tecnologico de Monterrey campus. Comparison showed that the total RMSE between the measurement campaign and the simulation approach was 2.07 dB, effectively capturing the environment propagation characteristics. Therefore, the obtained results, as well as the RF characteristics described in the scenario, share similarities with those commonly found in outdoor parking lots, aiding in the adequate characterization of the mmWave communication channel for new vehicular communications networks, applications, and deployments, considering outdoor conditions as well as the impact of different vegetation/vehicle densities, for current as well as for future wireless technologies. Although these promising mmWave channel characterization results help to facilitate the understanding of these complex outdoor environments, future research needs will require further studies in order to address challenges arising in the mmWave frequency range, such as heterogeneous networks, interference characterization, or the interconnection of myriad devices in wireless vehicular communication contexts beyond 5G.

## Figures and Tables

**Table 1:** RL algorithm simulation parameters.

Parameters	Values
TX Power	25 dBm
Carrier frequency	28 GHz
Bit Rate	4.62 Gbps
Antenna type/Gain	Directional/20 dB
3D Ray Launching: angular resolution/Rebounds	0.473 degree/3
Scenario size/Unitary volume analysis	(54 × 42 × 15) m/1m^3^ (1 × 1 × 1) m

## Data Availability

Not applicable.
